# Differentiating thyroid nodules parathyroid lesions using 2D-shear-wave elastography: a novel approach for enhanced diagnostic accuracy

**DOI:** 10.3389/fendo.2023.1231784

**Published:** 2023-07-31

**Authors:** Dana Stoian, Andreea Borlea, Laura Taban, Felix-Mihai Maralescu, Flaviu Bob, Oana Schiller, Adalbert Schiller, Octavian Neagoe

**Affiliations:** ^1^ Discipline of Endocrinology, Second Department of Internal Medicine, University of Medicine and Pharmacy “Victor Babes”, Timisoara, Romania; ^2^ Centre for Molecular Research in Nephrology and Vascular Disease, “Victor Babeş” University of Medicine and Pharmacy, Timişoara, Romania; ^3^ Clinic of Endocrinology, Timiş County Emergency Clinical Hospital, Timisoara, Romania; ^4^ Discipline of Nephrology, Second Department of Internal Medicine, University of Medicine and Pharmacy “Victor Babes”, Timisoara, Romania; ^5^ Dialysis Unit, Dialysis Medical Center B Braun Avitum, Timisoara, Romania; ^6^ Second Discipline of Surgical Semiology, First Department of Surgery, University of Medicine and Pharmacy “Victor Babes”, Timisoara, Romania

**Keywords:** hyperparathyroidism, thyroid nodules, elastography, 2D-SWE, neck ultrasonography, parathyroid adenomas

## Abstract

Differentiating between thyroid and parathyroid lesions by means of ultrasound can be a challenge in some cases. This study explores the diagnostic efficacy of bidimensional shear wave elastography planewave ultrasound (2D SWE PLUS) as an auxiliary technique in distinguishing these superficial structures. We evaluated 86 cases, presenting with concurrent thyroid nodules and hyperparathyroidism, through conventional ultrasound and 2D SWE PLUS, employing an Aixplorer Supersonic Mach30 with a 5-18 MHz linear probe. Statistically significant differences were observed for the elasticity index (EI) between parathyroid and normal thyroid tissue (p<0.0001, U=291), and between parathyroid lesions and thyroid nodules (p<0.0001, U=248.5). An area under the curve (AUC) of 0.961, with an optimal cut-off value of ≤8.9 kPa, was established to effectively distinguish parathyroid tissue from normal thyroid tissue (sensitivity of 91.9%; specificity of 97.5%). Furthermore, an AUC of 0.963 and an optimal cut-off of 9.24 kPa (sensitivity of 94.2%, specificity of 91.1%) were determined for parathyroid vs thyroid lesions. Elasticity values were significantly elevated in the cancer group compared to benign thyroid nodules (p<0.0001). Our findings suggest that 2D SWE PLUS is an effective tool in differentiating between thyroid nodules and parathyroid lesions, enhancing diagnostic performance in neck ultrasonography.

## 1 Introduction

High-resolution ultrasound (US) delivers exceptional anatomical detail for the superficial structures of the neck, making it the preferred initial imaging modality for evaluating neck masses. Consequently, it has become an indispensable element of clinical means of diagnosis and management of thyroid and parathyroid pathologies (1). This allows practitioners to capture accurate anatomical details and identify pathological lesions in most cases. Positioning the patient in a supine posture with the neck naturally extended facilitates better probe contact with the skin and enhances the ease of scanning the entire anterior cervical region (2).

In light of these advancements, diagnosing parathyroid disease *via* ultrasound evaluation continues to pose a challenge. In the past, it was thought that the key to successfully treating primary hyperparathyroidism was simply locating an experienced surgeon. However, in today’s era of minimally invasive surgeries—which offer reduced morbidity and comparable success rates—the crucial factor for treatment success lies in the precise pre-operative localization of the parathyroid lesion (3). Integrating cervical ultrasound and nuclear imaging, firstly 99mTechnetium-Sestamibi scintigraphy or in unclear cases adding single-photon emission computed tomography/computed tomography (SPECT/CT), is recognized as the predominant first-line evaluation strategy in the field. B-mode grayscale sonography is effective in identifying the position of a typical adenomatous parathyroid gland. However, detecting atypical intrathyroidal parathyroid adenomas remains a challenge, with sonography often failing in such cases. Ultrasound-guided fine-needle aspiration biopsy can also help differentiate intrathyroidal parathyroid glands from thyroid nodules (4, 5). There are also cases that associate both thyroid nodules and parathyroid adenomas or hyperplasia, making the diagnosis even more difficult (5). US-based methods, such as Color Doppler, different elastography methods and contrast-enhanced ultrasound (CEUS) are thought to aid the diagnosis and multiple studies focused their attention on developing a US-based multiparametric model (6). Nonetheless, current guidelines have yet to endorse multimodal evaluation, due to the lack of cost-effectiveness data and insufficient evidence supporting this approach.

Out of all additional US-based methods, elastography has gained a lot of confidence in detecting different thyroid pathologies, both nodular and diffuse (7–14). In our previous studies (15, 16), elastography demonstrated its accuracy as a predictor of parathyroid tissue compared to thyroid or muscle tissue, proving valuable for both primary and secondary hyperparathyroidism.

In bidimensional shear-wave elastography (2D-SWE), multiple focal zones are rapidly examined, generating a cylinder-shaped shear wave cone. This provides a real-time bidimensional monitoring of shear waves for estimating Young’s modulus of elasticity (E), based on the shear wave speed and illustrating quantitative elastograms. This technique offers the advantage of real-time visualization of a color quantitative elastogram overlaid on a B-mode image, in such way that the operator is provided both with anatomical images and tissue stiffness maps for diagnostic guidance (17). The primary aim of this study is to evaluate the efficacy of 2D-SWE as a non-invasive and accurate diagnostic tool for differentiating thyroid and parathyroid tissue. By assessing the variations in the elastic properties of these tissues, we aim to establish a reliable and objective criterion for their identification, ultimately enhancing diagnostic accuracy and facilitating better clinical decision-making in the management of thyroid and parathyroid disorders.

## 2 Materials and methods

### 2.1 Patients

**Figure 1:**
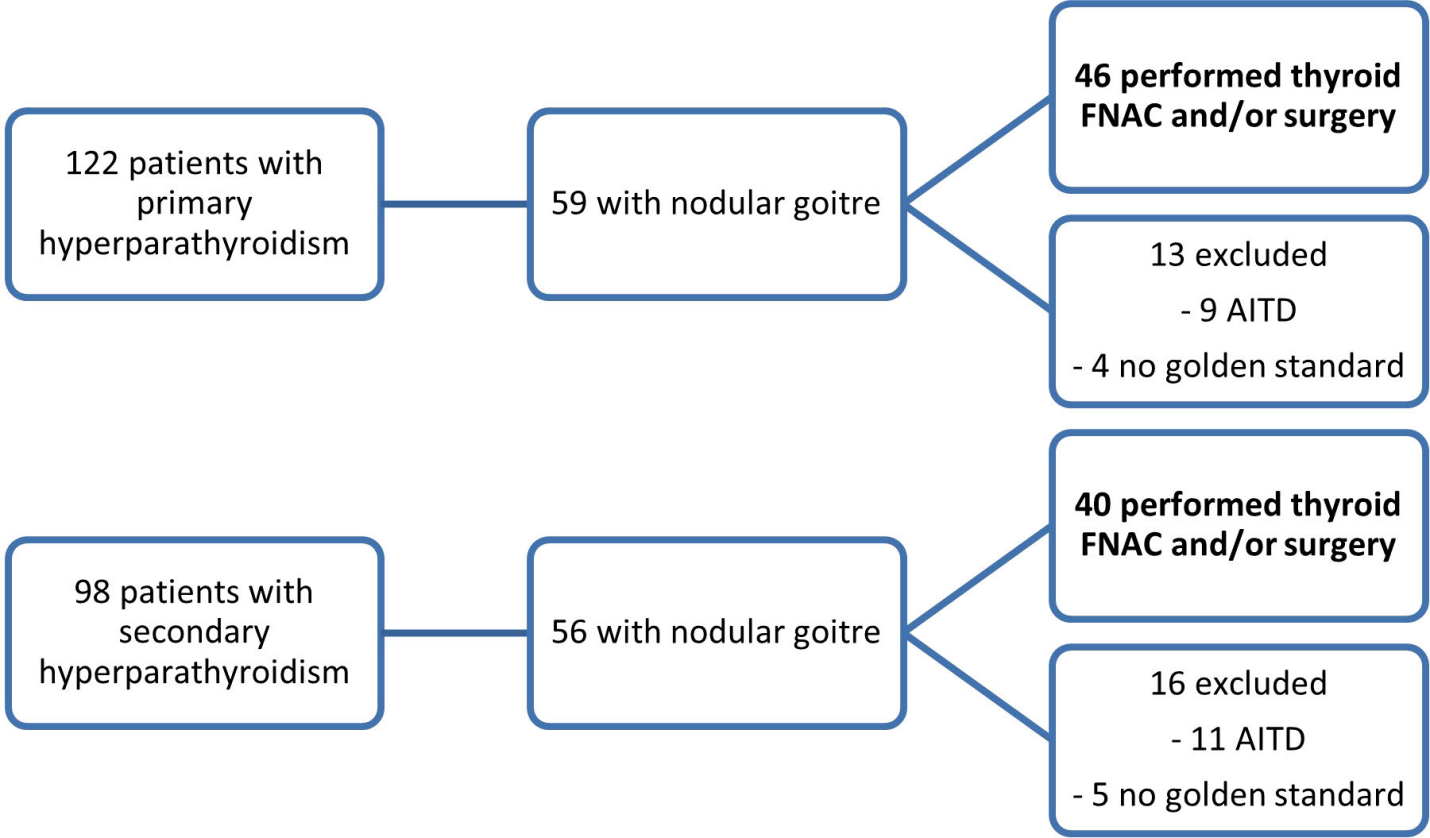
The inclusion of patients in the two study subgroups: patients with primary hyperparathyroidism and concomitant thyroid nodules and patients with secondary hyperparathyroidism and thyroid nodules (FNAC, fine-needle aspiration cytology; AITD, autoimmune thyroid disease).

In this prospective study, patients were recruited between January 2019 and September 2022. The selected patients presented concomitant thyroid nodules (one or more) and hyperparathyroidism (either primary or secondary to end stage renal disease), with US-detectable parathyroid tissue. Data was collected from patients evaluated at Dr. D Medical Center Timisoara and B Braun Dialysis Center Timisoara. Patients with previous surgery with parathyroid and/or thyroid involvement, patients with secondary hyperparathyroidism due to vitamin D deficiency and patients with concomitant autoimmune disease (Graves’ disease and chronic autoimmune thyroiditis) and patients with parathyroid glands that were not detectable by conventional ultrasound were excluded from our study. Of the 112 patients diagnosed with primary hyperparathyroidism who were initially evaluated, 46 had associated thyroid nodular disease. Only the cases with cytology or pathology report were included in our study. Ninety-eight patients with secondary hyperparathyroidism were identified, out of which 40 patients associated nodular thyroid disease with cytology or pathology evaluation. **Figure 1** details the selection of patients. The study was approved by the University of Medicine and Pharmacy “Victor Babes” Timisoara Scientific Ethics Committee (UMFT CECS Nr.32/28.09.2018), and written informed consent was obtained from all patients. The study followed the Ethics Code as stated by the Declaration of Helsinki, amended in 2000, Edinburgh.

### 2.2 Conventional US evaluation

The patient was positioned supine, with the neck extended, for the ultrasound examination, with coupling gel inserted between the transducer and the skin on the neck. All examinations were carried out by a US operator with more than 10 years of thyroid US experience and more than 4 years of thyroid SWE expertise. The Aixplorer Mach 30 machine (Hologic, Aix-en-Provence, France) was used for all patients’ examinations, utilizing a linear, high-resolution transducer of 18-5 MHz. The measurements were carried out by viewing the thyroid lobe in longitudinal or transverse plane and the depth was set to encompass the complete thyroid lobe in the center of the picture on the display in B-mode. Where possible, images of both thyroid nodule and parathyroid tissue were obtained in the same frame. In cases where the two lesions were impossible to capture in one frame, different frames were obtained for each lesion. The location, margins, echogenicity, presence of hyperechoic foci, shape were described and used in the risk-stratification of each thyroid nodule, according to the Thyroid Imaging and Reporting Data System (TIRADS) elaborated by Russ and collaborators (18). For parathyroid adenomas, the location was mentioned in each case.

### 2.3 Elastography evaluation

**Figure 2:**
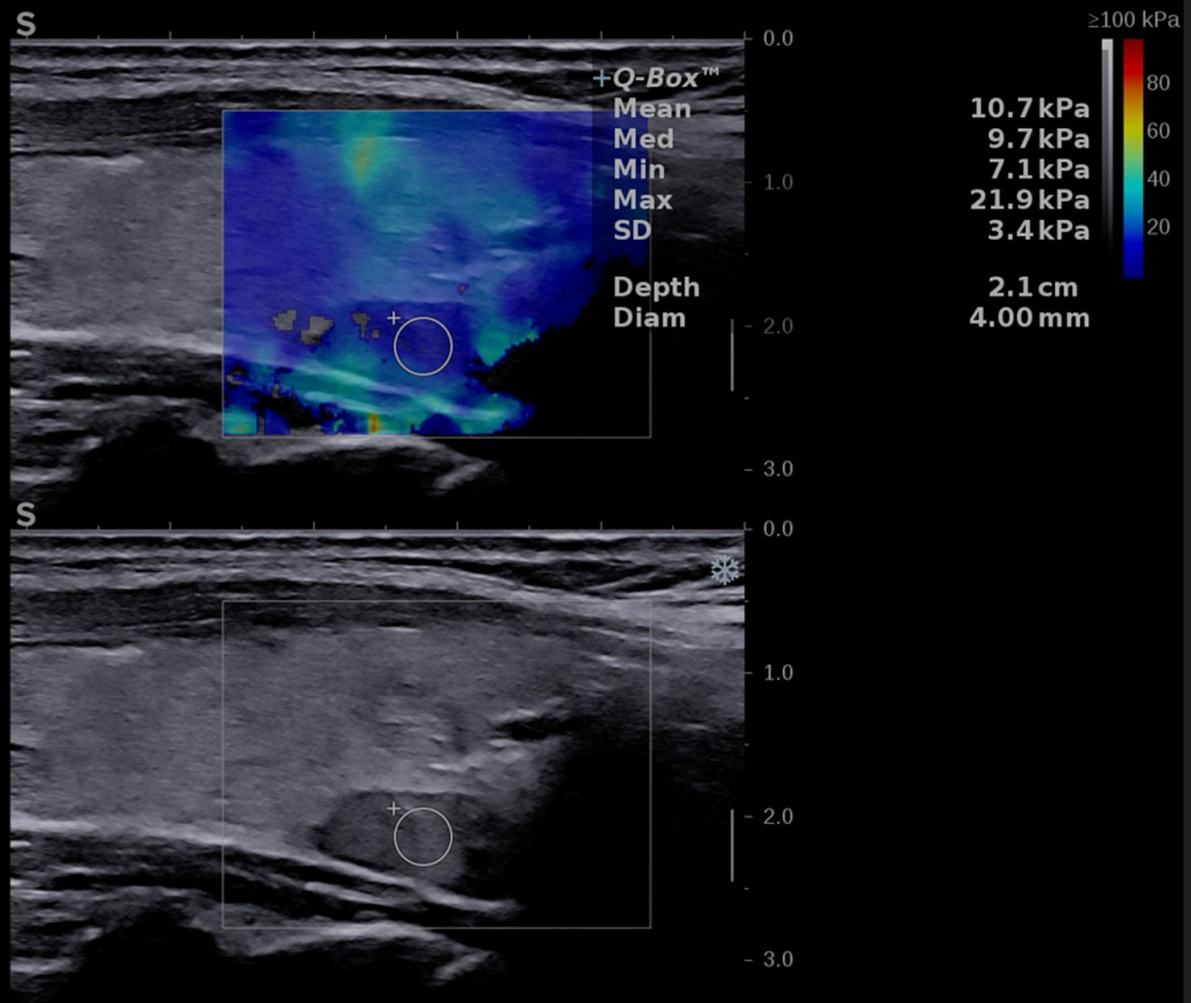
Conventional ultrasound image (displayed in the lower part of the image) showing an oval-shaped, hypoechoic, homogeneous mass located behind the left thyroid lobe. Shear-wave elastography image (shown in the upper part of the image) identifying a soft lesion, with a mean elasticity index (EI) of 10.7 kPa.

**Figure 3:**
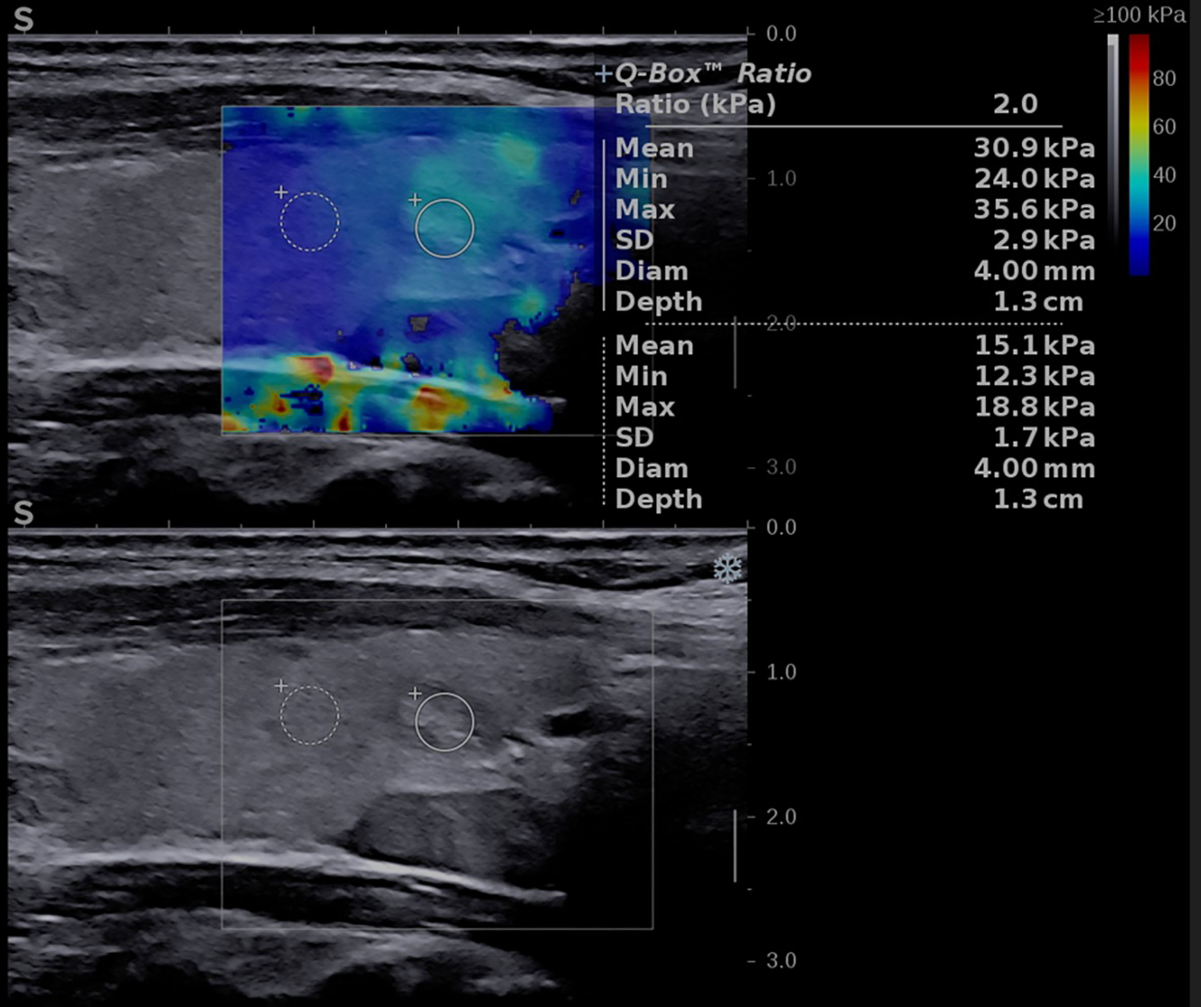
Conventional ultrasound image (displayed in the lower part of the image) showing an oval-shaped, well-delimitated, solid, inhomogeneous mass located in the left thyroid lobe. Shear-wave elastography image (shown in the upper part of the image) identifying a soft lesion, with a mean elasticity index (EI) of 30.9 kPa.

The elasticity of the thyroid nodule and parathyroid tissue was estimated using 2D shear-wave elastography plane-wave ultrasound (2D-SWE PLUS) available on the Aixplorer Mach 30 device, using the same linear transducers mentioned above. The evaluation was performed immediately after the conventional US, by the same operator. The examiner applied no manual compression on the probe for at least 6 seconds, maintaining probe contact with the skin. The transducer generates acoustic vibrations towards the thyroid and parathyroid tissue to estimate tissue elasticity. After image stabilization, the device provides a real-time elastogram displayed as a color-map that is overlaid on the greyscale image. A stable image was frozen by the operator, who selected a region-of-interest (ROI) and placed the quantification box (Q-box), sized between 5 and 10 mm, at the level of the targeted lesion. Additionally, to the elastogram, a quantitative measure is provided: the mean, median (Med), minimum (Min), and maximum (Max) values of the elasticity index (EI), measured in kilopascals (kPa) together with the standard deviation (SD and the depth of the measurement (see **Figure 2** -parathyroid evaluation and **Figure 3** – thyroid nodule evaluation). The scale was set between 0 and 100 kilopascals, as indicated for thyroid evaluation. For each evaluated lesion, three measurements were performed, and the median value was considered in the analysis. For thyroid nodules, the SWE results were also considered in the TIRADS risk-stratification. For each nodule we performed 5 measurements and considered the median values in the analysis.

### 2.4 Statistical analysis

Statistical analysis was conducted utilizing MedCalc V19.4 software (MedCalc Software, Belgium). Demographic and clinical data, in addition to ultrasonography findings, were characterized using descriptive statistics. Normality of numerical variables was assessed *via* the D’Agostino-Pearson test. Numerical variables demonstrating normal distributions were described using mean and standard deviation, while non-normally distributed variables were depicted using median and interquartile range (IQR) of 25-75%. Qualitative variables were represented with figures and percentages. To differentiate between non-parametric variables, the Mann-Whitney U test was employed, while parametric variables were analyzed using the parametric t-test. Data representation and comparison of median values were facilitated through the generation of box plots, which provided a comprehensive visual summary of the central tendency and dispersion of the dataset. Further, to evaluate the diagnostic performance of SWE for detecting thyroid and parathyroid tissues, receiver operating characteristic (ROC) curves were utilized. This analysis enabled the establishment of an optimal cut-off value to distinguish between these two tissue types. A p-value threshold of 0.05 was considered to denote statistical significance.

### 2.5 Institutional review board statement

The study was conducted in accordance with the Declaration of Helsinki and approved by the Scientific Ethics Committee of the University of Medicine and Pharmacy “Victor Babes” Timisoara (UMFT CECS Nr.32/28.09.2018). Informed consent was obtained from all subjects involved in the study.

## 3 Results

### 3.1 Baseline characteristics of the entire study group

A total of 86 patients were finally included in the analysis, with concomitant thyroid and parathyroid disease without associated autoimmune thyroid disease. Twenty-nine were man and fifty-seven were women. The baseline characteristics of the group, and by contrast in the primary and secondary hyperparathyroidism groups, are detailed in **Table 1**. Normally distributed variables are presented as mean ± SD and variables with non-normal distribution are presented as median (range).

**Table 1:** Baseline characteristics of the entire group and in the two hyperparathyroidism subgroups: primary and secondary form of disease.

Parameter		Study group	PHPT	SHPT	p
Age (years)		57.6 ± 12.4	57 ± 13.2	58 ± 11.5	0.6430
TSH (mUI/L)		3.3 ± 1.2	3 ± 1.31	3.6 ± 1.2	0.0643
PTH (pg/mL)		254.5 (94.1-3720)	166.8 (94.1-1493)	773.5 (200.7-3720)	<0.0001
Total Calcium (mg/dL)		9.7 ± 1.3	10.4 (9-13.4)	8.9 (6.2-9.9)	<0.0001
Vitamin D (ng/mL)		25.3 (7-55.3)	21.3 (10.8-46.7)	35.8 (7-55.3)	<0.0001
Mean EI normal thyroid (kPa)		12.2 (7.2-23.8)	11.5 (7.2-23.8)	12.6 (9-19.1)	0.3343
Min EI normal thyroid (kPa)		8.8 ± 2.9	8.5 ± 3.2	9 ± 2.5	0.2495
Max EI normal thyroid (kPa)		18 (9.9-42.5)	18.2 (9.9-42.5)	17.5 (11.9-30.4)	0.8796
Mean EI parathyroid (kPa)		5.8 (2.3 – 21.3)	4.6 (2.3-8.9)	6.9 (3.9-21.3)	<0.0001
Min EI parathyroid (kPa)		2.5 (0.1 – 15.5)	0.4 (0.1-5.3)	3.8 (1.3-15.5)	<0.0001
Max EI parathyroid (kPa)		10.9 (5.3-29.4)	10.5 (5.3-17.4)	11.3 (5.9-29.4)	0.2678
Mean EI TN (kPa)		17 (5.9-65)	15 (7.7-54.3)	20.6 (5.9-65.7)	0.0014
Min EI TN (kPa)		12.4 (4.1-63)	8.1 (2.2-16.8)	8.6 (3.9-14.9)	0.0003
Max EI TN (kPa)		26.1 (10.4-107.5)	18.2 (9.9-42.5)	17.5 (11.9-30.4)	0.0001
TN/parathyroid ratio		3.2 (0.7-10)	3.4 (1.4-7.7)	3 (0.6-10)	0.3365
TIRADS	2	1 (1%)	1 (2%)	0 (0%)	0.396
	3	18 (21%)	17 (37%)	1 (2%)	0.0002
	4a	31 (36%)	12 (26%)	19 (48%)	0.058
	4b	25 (29%)	11 (24%)	14 (35%)	0.377
	5	11 (13%)	5 (11%)	6 (15%)	0.818

In the group with primary hyperparathyroidism, we detected one adenoma in all cases. In the secondary hyperparathyroidism (SHPT) group, the median number of US-detectable enlarged glands was 2 (range between 1 and 4).

### 3.2 Elastography measurements

**Figure 4:**
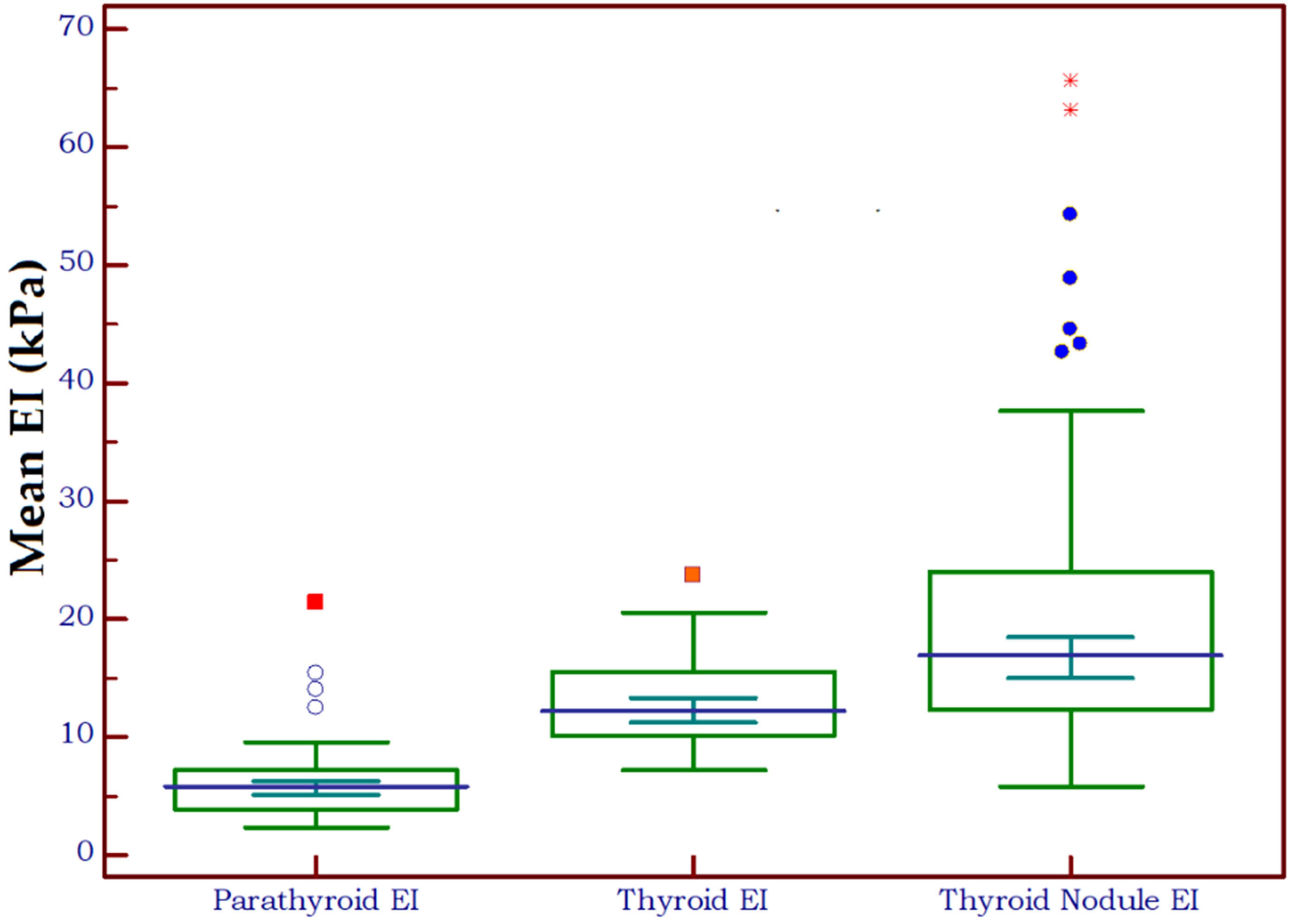
Box-and-whisker distribution plot illustrating the 2D-SWE PLUS elasticity index (EI) in three different tissue types: parathyroid tissue, normal thyroid tissue and thyroid nodules.

**Figure 4** shows the distribution of EI values in three different types of tissue: parathyroid tissue, normal thyroid tissue and thyroid nodules. Statistically significant differences were detected both between parathyroid tissue and normal thyroid (p<0.0001, U=291) and between parathyroid and thyroid nodules (p<0.0001, U=248.5).

There were significant differences in terms of the mean EI between the primary and secondary hyperparathyroidism (p<0.0001).

**Figure 5:**
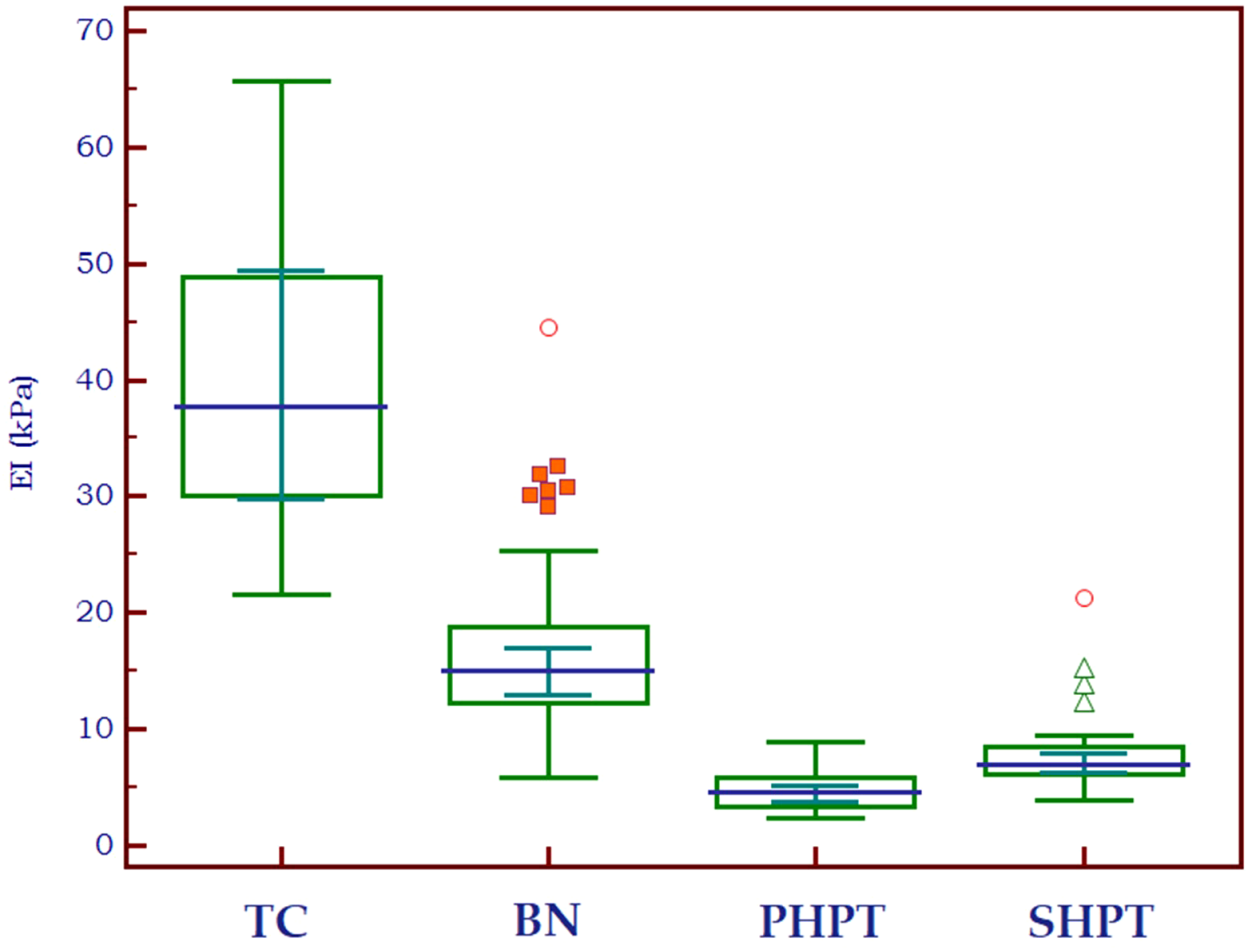
Mean 2D SWE PLUS values displaying differences between thyroid cancer, benign thyroid nodules, primary and secondary hyperparathyroidism (TC, thyroid cancer; BN, benign nodule; PHPT, primary hyperparathyroidism; SHPT, secondary hyperparathyroidism EI, elasticity index).

The elasticity index of thyroid nodules (benign and malignant) and parathyroid lesions (primary and secondary are compared in **Table 2** to the value of normal thyroid tissue, with statistically significant differences in all cases. **Figure 5** displays the median elasticity index for all of the aforementioned groups.

**Table 2:** Differences in elasticity compared to normal thyroid tissue.

Parameter	Entire group	p
*Mean EI TC (kPa)*	37.65 (30.10 – 48.86)	< 0.0001
*Mean EI BN (kPa)*	15.06 (12.22-18.80)	0.0010
*Mean EI PHPT (kPa)*	4.64 (3.34-5.82)	< 0.0001
*Mean EI SHPT (kPa)*	6.91 (6.09-8.44)	< 0.0001
*Mean EI Thy (kPa)*	12.2 (7.2-23.8)	–

TC, thyroid cancer; BN, benign nodule; PHPT, primary hyperparathyroidism; SHPT, secondary hyperparathyroidism; Thy, normal thyroid tissue.

### 3.3 2D-SWE PLUS in differentiating thyroid from parathyroid tissue

**Figure 6:**
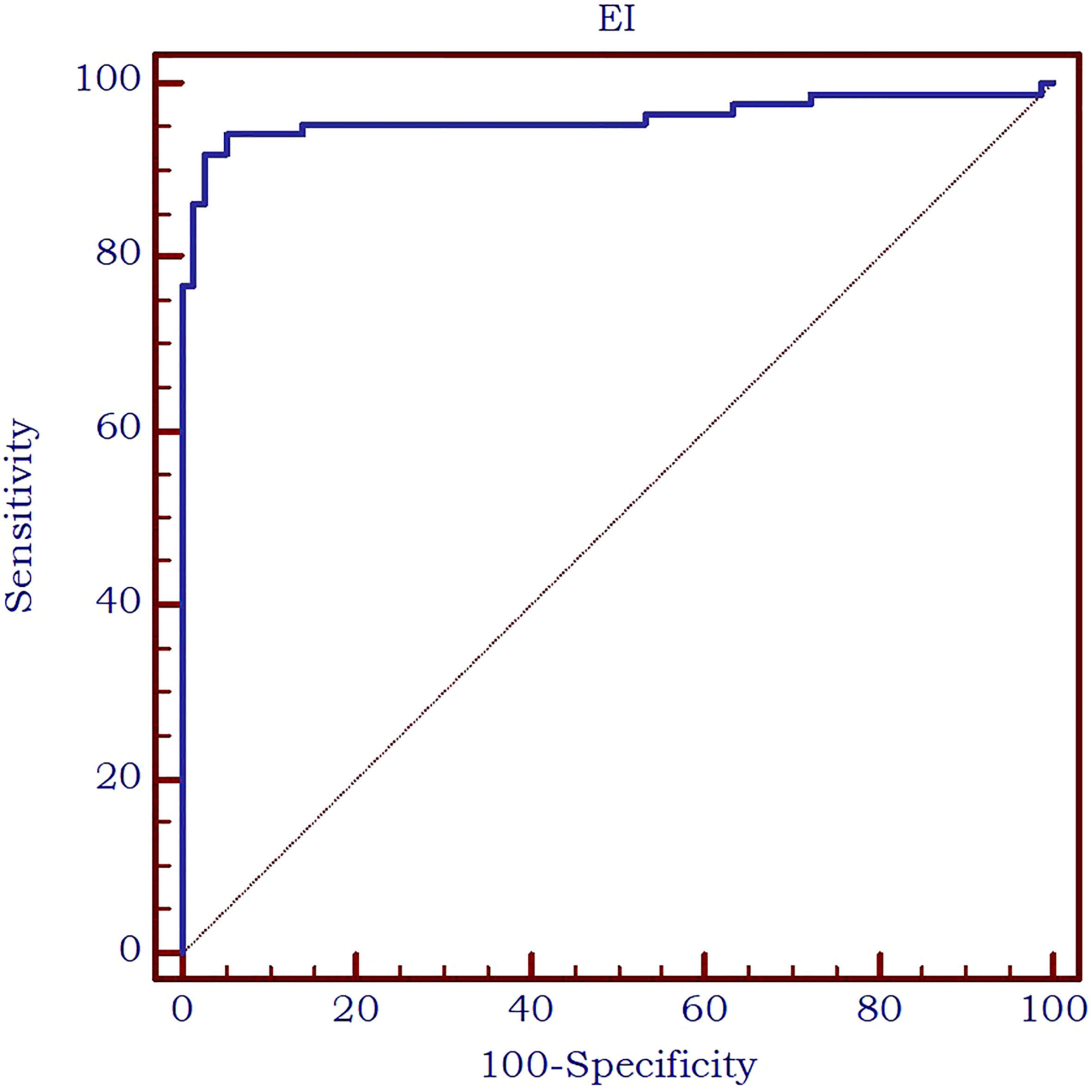
AUC for the performance of 2D-SWE PLUS in differentiating thyroid and parathyroid tissue.

The analysis of the receiver operating characteristic (ROC) curve demonstrated an excellent value of the area under the curve (AUC) value of 0.961 and an optimal cut-off value of ≤8.9 kPa was determined to effectively differentiate between parathyroid tissue and normal thyroid tissue. The diagnostic performance metrics at this cut-off value were as follows: sensitivity of 91.9%, specificity of 97.5%, positive predictive value (PPV) of 97.5%, and negative predictive value (NPV) of 91.7%. The AUC is displayed in **Figure 6**.

### 3.4 2D-SWE PLUS in differentiating between thyroid nodules and parathyroid glands

**Figure 7:**
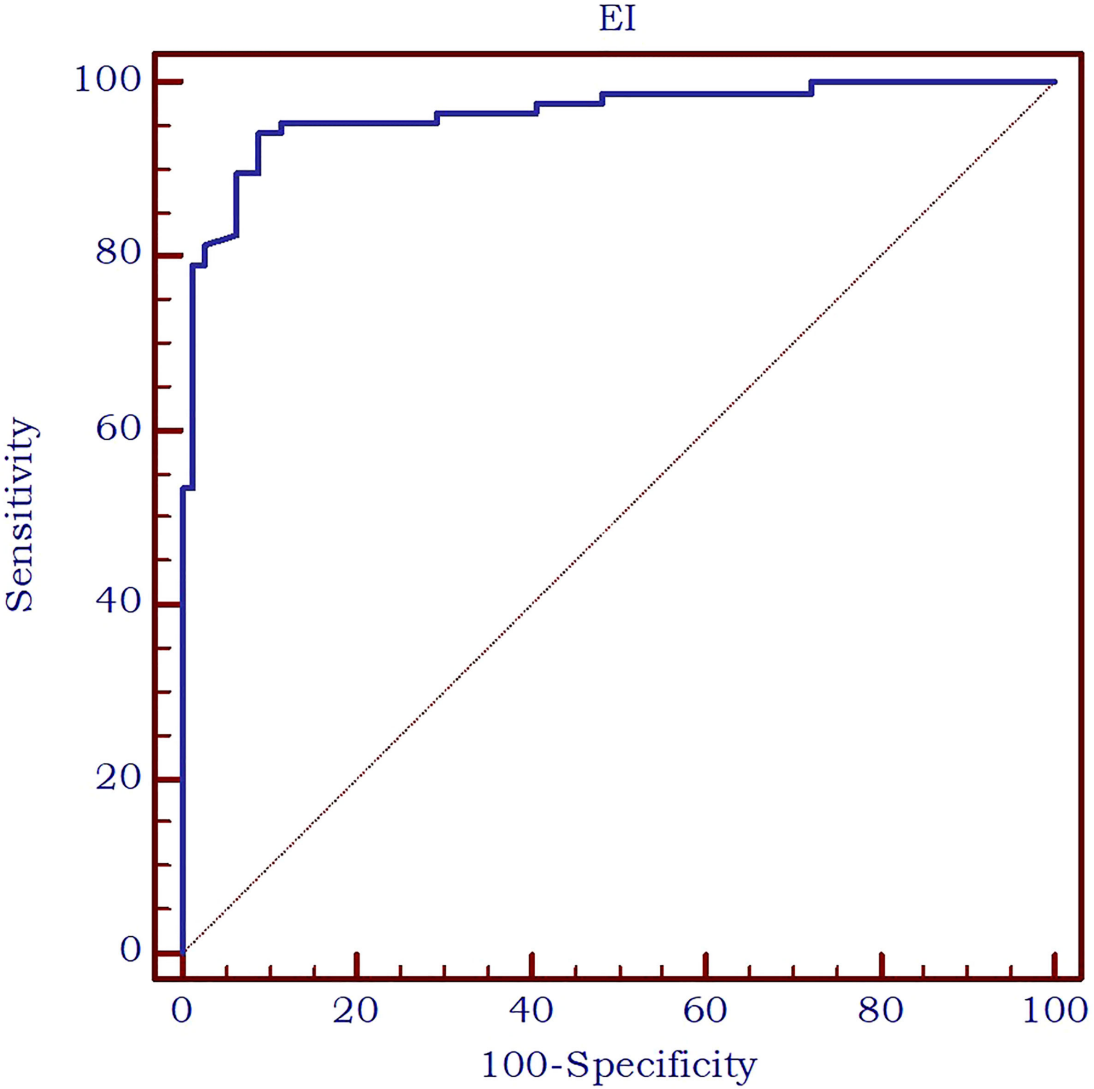
AUC for the performance of 2D-SWE PLUS in differentiating thyroid nodules and parathyroid enlargement or adenomas.

Furthermore, the AUC exhibited an excellent value of 0.963 (**Figure 7**) in differentiating between thyroid nodules and enlarged parathyroid glands or adenomas. A cut-off value for 2D SWE PLUS technique has been established at ≤9.24 kPa for the discrimination of parathyroid and thyroid nodules. The diagnostic performance at this threshold demonstrated a sensitivity of 94.2%, specificity of 91.1%, negative predictive value (NPV) of 93.5%, and positive predictive value (PPV) of 92%.

### 3.5 Correlations between tissue elasticity and patient-related parameters

Thyroid nodule elastography indices were correlated positively and strongly with the presence of malignancy (r=0.729, p<0.0001), weakly with parathyroid lesion elasticity (r=0.208, p=0.0142) and PTH (r=0.357, p=0.0007). Parathyroid lesion EI were correlated negatively and weakly with serum calcium values (r=-0.380, p=0.0003) positively and weakly to PTH values (r=0.226, p=0.0364). All correlations are displayed in **Table 3**.

**Table 3:** Correlations between thyroid and parathyroid 2D-SWE and clinical and biochemical parameters.

		Calcium	Pathology	EI PT	EI TN	TSH	Age	Vit D
*Calcium*	r p n	–						
*Pathology*	r p n	-0.049 0.6519 86	–					
*EI PT*	r p n	-0.380 0.0003 86	0.208 0.0551 86	–				
*EI TN*	r p n	-0.147 0.1772 86	0.729 <0.0001 86	0.264 0.0142 86	–			
*TSH*	r p n	-0.211 0.0514 86	0.137 0.2077 86	0.164 0.1314 86	0.111 0.3075 86	–		
*Age*	r p n	0.123 0.2603 86	-0.157 0.1486 86	-0.178 0.1019 86	-0.156 0.1512 86	0.018 0.8670 86	–	
*Vit D*	r p n	-0.242 0.0248 86	0.085 0.4340 86	0.167 0.1250 86	0.198 0.0677 86	0.181 0.0952 86	0.052 0.6321 86	–
*PTH*	r p n	-0.214 0.0476 86	0.227 0.0356 86	0.226 0.0364 86	0.357 0.0007 86	0.030 0.7830 86	0.000 0.9973 86	0.207 0.0561 86

### 3.6 Thyroid cancer cases

Upon examination of the cytological and pathological reports of the 86 analyzed thyroid nodules, 72 cases were classified as benign, while 14 were malignant. There were 10 cancers in the SHPT group and 4 in the PHPT group. A detailed analysis of the malignancies revealed the following distribution: 11 cases of papillary thyroid cancer (PTC), consisting of 9 macrocarcinomas and 2 microcarcinomas; one case of medullary thyroid cancer (MTC); one case of follicular thyroid cancer (FTC); and one case of non-invasive follicular thyroid neoplasm with papillary-like nuclear features (NIFT-P). We characterized the NIFT-P case together with the malignancies, given its slightly increase EI compared to benign tissue. Elasticity index values were significantly increased in the cancer group compared to benign thyroid nodules (p<0.0001).

**Figure 8:**
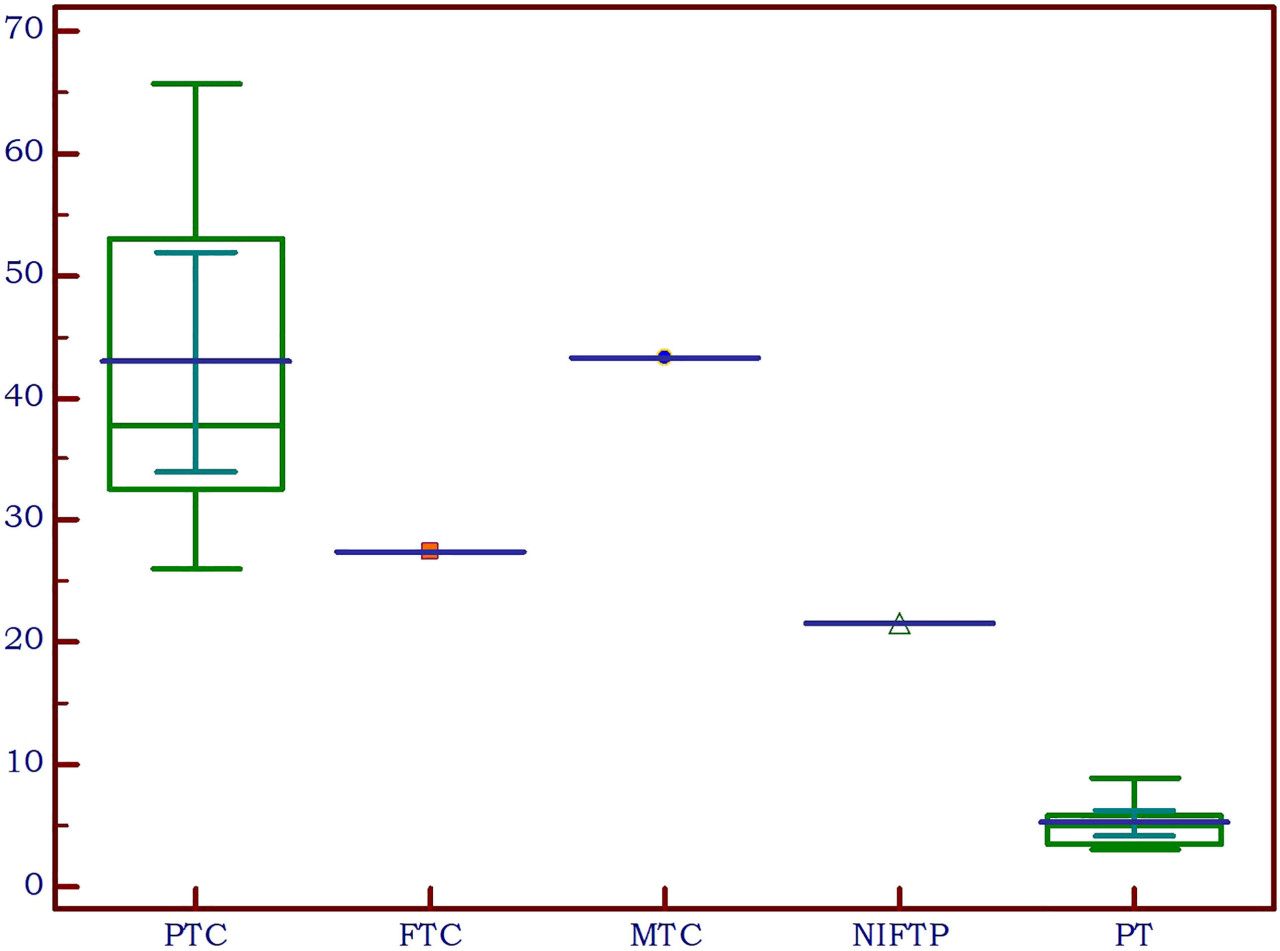
Thyroid cancer pathology in the study group compared to parathyroid lesions (PTC, papillary thyroid cancer; FTC, follicular thyroid cancer; MTC, medullary thyroid cancer; NIFTP, non-invasive follicular thyroid neoplasm with papillary-like nuclear features; PT, parathyroid lesion).

Considering the various thyroid cancer subtypes identified within our dataset, we aimed to assess if there are significant differences between these cancer subgroups in comparison to parathyroid tissue. Given that none of the thyroid malignancies recorded an EI value lower than the cut-off value established above for parathyroid tissue, we did not conduct separate comparisons for cases of primary and secondary hyperparathyroidism. A value less than the predetermined cut-off of 9.24 kPa was noted in only 7 cases with thyroid nodules, representing 8.1% of the total. Notably, all these nodules were benign. A significant difference was detected between papillary thyroid cancer and parathyroid lesion (p<0.0001). Given the limited sample size, with only one case each for follicular thyroid cancer, medullary thyroid cancer, and NIFT-P, it was not feasible to conduct a comparison of statistical significance between these subgroups and parathyroid lesions. The values for each subtype are detailed in **Table 4** and illustrated in **Figure 8**.

**Table 4:** Thyroid cancer subtypes compared to parathyroid lesions.

	n	Median (25-75 IQR)	p
*PTC*	11	37.7 (32.5-52.9)	<0.0001
*FTC*	1	43.3	–
*CMT*	1	27.4	–
*NIFT-P*	1	21.6	–
*Parathyroid lesion*	14	5 (3.5-5.8)	–

## 4 Discussion

The simultaneous occurrence of thyroid nodular disease and parathyroid lesions due to primary or secondary hyperparathyroidism can complicate the process of clinical diagnosis and treatment for those affected. The reported incidence of concurrent thyroid nodules in patients with primary hyperparathyroidism varies widely in scientific literature, ranging from 18% to 85% [ (19–22) and about 62% (23) in patients with secondary hyperparathyroidism in end-stage renal-disease. The exact relationship between PHPT and thyroid disease is not fully understood and is likely multifactorial (21). The exact identification of the diseased parathyroid tissue utilizing preoperative imaging techniques is essential for surgery to be effective. Due to the introduction of some cutting-edge problem-solving techniques in recent years, a multiparametric US approach has been adopted. This has increased the ability of lesion characterization, improved diagnostic accuracy, and led to an increased importance of US utilization in clinical decision. CEUS and elastography, in addition to the conventional B-mode greyscale and Color Doppler US, are included in the multimodal US-based pathway (24–26).

In this study, we have presented a novel approach to differentiating parathyroid lesions from thyroid nodules using 2D Shear-Wave Elastography (2D-SWE) Plane-Wave US, aiming to enhance the diagnostic accuracy in the management of these conditions. We chose to exclude cases with concomitant autoimmune thyroid disease in order not to have falsely increased elastography values for thyroid tissue (27). Our findings suggest that 2D-SWE provides a valuable tool in distinguishing between these two types of lesions, offering potential benefits in both diagnostic accuracy and patient management.

Our earlier research has confirmed that the elasticity of parathyroid tissue is inferior to that of thyroid tissue (15, 16). The current study results are in concordance with these results. We chose to use for comparisons the mean EI as the SWE parameter, as it was established to be the most effective metric for delivering quantitative elasticity data on parathyroid adenomas (15).

Research literature in the domain of primary hyperparathyroidism has identified varying threshold values for parathyroid adenomas, specific on the elastography techniques employed. In our previous studies, a cut-off of 9.58 kPa was established between parathyroid tissue and normal thyroid tissue (15). By utilizing Shear Wave Virtual Touch Imaging Quantification, higher values were determined for parathyroid adenomas (2.16 ± 0.33 m/s) as compared to the values for parathyroid hyperplasia (1.75 ± 0.28 m/s), setting a cut-off value exceeding 1.92 m/s for parathyroid adenomas (28). With the identical elastography method, another investigation made a comparison between parathyroid adenomas and thyroid tissue, concluding that the shear wave velocity value for parathyroid adenoma was less than that of thyroid tissue (2.01 m/s and 2.77 m/s, respectively) (29). In the current study, we detected a cut-off of below 8.9 kPa for parathyroid versus thyroid tissue, regardless of the etiology. As our aim was to differentiate between nodular lesions arising from thyroid or parathyroids, we detected an optimal cut-off of below 9.24 kPa for differentiating thyroid nodules and parathyroid lesions, with most of the primary hyperparathyroidism cases (25-75% IQR) displaying values between 3.3-5.8 kPa, secondary hyperparathyroidism between 6-8.4 kPa, the majority of benign thyroid nodules displaying values between 12.2-18.8kPa and most malignant thyroid nodules between 30.1-48.8 kPa.

Moreover, there were statistically significant differences observed between the mean EI SWE values of in primary and secondary hyperparathyroidism (p<0.001). This observation aligns with the findings from previous studies (15, 19). Conventional US characteristics and Color Doppler US are not able to differentiate alone between adenoma and hyperplasia. In B-mode, pathological parathyroids may appear as oval-shaped lesions that are enlarged, confined, hypoechoic, and bordered by hyperechoic connective tissue (6). Both adenoma and hyperplasia are typically shown on the Color Doppler with significant feeding polar arteries which enter the pole and extend along the edge of the parathyroid gland (30).

The prevalence of thyroid malignancy in our group was higher in the SHPT group (10/40, 25% of the nodules) compared to the PHPT group (4/46, 8.7% of the nodules). This could explain the significantly higher thyroid nodule EI between the PHPT and the SHPT subgroups. However, in both groups the incidence of thyroid cancer was significantly higher comparative to the prevalence of cancer in thyroid nodules in general, which is widely variable in the literature, but it is considered to be around 5% (31–34).

Although this was not the aim of the current study, we found a significant increase in the EI values among malignant cases compared to benign thyroid nodules, which confirms the excellent role of elastography in differentiating malignant from benign thyroid lesions. These findings are in harmony with previous studies demonstrating that malignant thyroid nodules tend to exhibit higher stiffness on elastography compared to benign nodules (35–39).

## 5 Conclusions

The use of the established cut-off values for 2D-SWE, as identified in our analysis, offers promising avenues for improving diagnostic specificity and sensitivity in distinguishing between thyroid nodules and parathyroid lesions. The ability to distinguish these lesions effectively and non-invasively could considerably impact the clinical approach, potentially reducing diagnostic costs, evaluation time, invasive procedures and improving patient outcomes.

## Data availability statement

The original contributions presented in the study are included in the article/supplementary material. Further inquiries can be directed to the corresponding author.

## Ethics statement

The studies involving human participants were reviewed and approved by the University of Medicine and Pharmacy “Victor Babes” Timisoara Scientific Ethics Committee. The patients/participants provided their written informed consent to participate in this study.

## Author contributions

Conceptualization, DS and AB; methodology, DS; software, F-MM; validation, AS, OS and ON; formal analysis, ON and FB; investigation, DS and LT; resources, DS; data curation, AB and F-MM; writing—original draft preparation, AB and DS; writing—review and editing, AB; visualization, LT and FB; supervision, DS; project administration, AB. All authors have read and agreed to the published version of the manuscript. 
